# Measuring Outcome after Wrist Injury: Translation and Validation of the Swedish Version of the Patient-Rated Wrist Evaluation (PRWE-Swe)

**DOI:** 10.1186/1471-2474-12-171

**Published:** 2011-07-22

**Authors:** Cecilia Mellstrand Navarro, Sari Ponzer, Hans Törnkvist, Leif Ahrengart, Gunnar Bergström

**Affiliations:** 1 Department of Clinical Science and Education, Orthopeadics Section, Södersjukhuset, Karolinska Institutet, Stockholm, Sweden; 2 Department of Public Health Sciences, Division of Intervention and Implementation Research, Karolinska Institutet, Stockholm, Sweden

**Keywords:** Wrist fracture, distal radius, cross-cultural adaptation, validity, DASH, PRWE

## Abstract

**Background:**

There is a need for outcome measurement instruments for evaluation of disability after trauma. The Patient-Rated Wrist Evaluation (PRWE) is a self-administered region-specific outcome measuring instrument developed for use in evaluating disability and pain of the wrist. The aim of this study is to translate and to cross-culturally adapt the PRWE for use in a Swedish patient population. Moreover, we aim at investigating the PRWE in terms of validity, reliability and responsiveness.

**Methods:**

We performed a translation and cross-cultural adaptation of the PRWE to Swedish (PRWE-Swe), utilising the process recommended by the American Association of Orthopedic Surgeons. A total of 124 patients with an injury to the wrist were included in the study. They filled in the PRWE and the DASH questionnaires at two separate occasions.

**Results:**

Reliability of the PRWE in terms of internal consistency (Cronbach's alpha 0.97) and test-retest stability (intraclass correlation coefficient 0.93) were excellent. Face validity and content validity were judged as good. Criterion validity assessed as the correlation between the PRWE and the DASH was also good (Spearman's rho = 0.9). Responsiveness measured by the standardized response mean (SRM) was good with an SRM_PRWE _of 1.29.

**Conclusion:**

This Swedish version of the PRWE is a short and easily understood self-administered questionnaire with good validity, reliability, and responsiveness. Our results confirm that the PRWE is a valuable tool in evaluating the results after treatment of a wrist injury.

## Background

The outcome after treatment of musculo-skeletal injuries and diseases has traditionally been measured by the range of motion, muscle strength, radiographic appearance, as well as the subjective judgement of the examiner. However, these traditional outcome measurements are not clearly correlated with how the patient assesses the end result. Over the last decades patient-based self-report instruments have been created to evaluate function and disability after disorders of different parts of the musculo-skeletal system [1].

In the field of upper limb injuries and diseases, the Disabilities of the Arm, Shoulder and Hand (DASH) questionnaire has evolved as one of the most important self-reported instruments [2-5]. It has been adapted and validated for use in Swedish language [5]. It has been proven to be useful in evaluating shoulder and elbow problems, as well as in follow-up of injuries and surgery to the wrist [6,7]. Subsequently, the Patient-Rated Wrist Evaluation (PRWE) questionnaire was developed in 2006. The PRWE is specifically designed to reflect the function of the wrist, in opposition to the DASH that takes the whole upper extremity into account. Studies have found the PRWE to be a valid and responsive questionnaire regarding wrist function [7,8]. Furthermore, one study claims that the PRWE should be preferred over the DASH when assessing wrist function, since it contains fewer items than the DASH and is quicker and easier for the patient to fill out [9].

The purpose of this study is to translate, adapt and validate the PRWE for use in Swedish language, by applying the adaptation process recommended by the American Association of Orthopedic Surgeons [10,11]. Moreover, the study aims to confirm that the Swedish version of the PRWE is as valid and responsive as the DASH for evaluating function after wrist injuries. When this study was initiated and conducted, the PRWE had not yet been validated for use in a Swedish patient population.

This study was planned according to International Conference on Harmonization guidelines for good clinical practice and it was approved by the local Ethics Committee in Stockholm (Ref No. 2007/1351-31/1).

## Methods

### The PRWE questionnaire

The PRWE was originally described by MacDermid et al. in 1996 [8,12,13]. The aim of the questionnaire was to provide a reliable and valid tool for quantifying patient-rated wrist pain and disability in order to assess outcome in patients with a distal radius fracture. The questionnaire was developed by surveying international wrist experts, reviewing the biomechanical literature and carrying out patient interviews. The final PRWE questionnaire consists of 15 questions divided into two subscales assessing pain and function. The pain subscale includes five items and the function subscale ten items. The questions are scored on a 10-point ordered categorical scale ranging from no pain or no difficulty (0 points) to worst pain or unable to do (10 points). The pain score is the sum of five items, a worst score of 50. The function score is the sum of ten items divided by two. The pain and the function scores are then summed. Thus, the PRWE ranges from 0 (a perfectly well functioning wrist free of pain), to a total of 100 (a completely disabled and painful wrist) [13].

### The adaptation process

The first stage of our adaptation process was a translation of the Canadian version of the PRWE by two independent bilingual translators with Swedish as their mother tongue. Both translators were medically trained but only one was fully informed of the aim of the use of the questionnaire and the translation. The two translators met to discuss the differences between the two forward translations. This discussion was witnessed by a senior orthopaedic surgeon. Differences were resolved by consensus between the two translators. This version of the Swedish PRWE was given to two independent native English translators for back translation into English. One of these translators was medically trained but was not aware of the purpose of the translation or the use of the questionnaire. The other translator was a professional translator with no medical training and no information regarding the purpose of the translation. The translations were reviewed in a meeting attended by one of the forward translators, one of the back translators, one outcome methodologist, one research nurse, and three medically trained experts from the field of orthopeadics. Differences were resolved by consensus. A prefinal version was created and field-tested on 18 healthy individuals and 32 patients with a variety of orthopaedic injuries. The final version (Additional file 1) was made after revising the prefinal version according to the comments from the healthy individuals and patients after field-testing.

In order to establish a reliable questionnaire, this final version was subsequently tested for *reliability*, *validity *and *responsiveness*.

### Reliability

Reliability describes the ability of an instrument to yield consistent and reproducible results. The degree of reliability was evaluated by analysing the *internal consistency *and the *test-retest stability*.

#### Internal consistency

This item of a scale concerns the association of the included items with one another [14]. Items constructed to assess a single underlying continuum should yield consistent responses, and the scale items should correlate highly with one another. Cronbach's alpha coefficient is applied to estimate the internal consistency [15].

#### Test-retest stability

This item is defined as the consistency of scores over time among respondents with a clinical status assumed not to be changed. It is tested when the instrument is administered two or several times during a period when no clinical change occurs. The results should be consistent at all measurement points. Test-retest stability is assessed with the use of the intraclass correlation coefficient [16]. The value should reach >0.75 for the instrument to be considered stable [6]. The intraclass coefficient takes into account the actual magnitude of scores and the agreement between ratings, not only the correlation and linear association among variables. For the test-retest stability analysis, the patients with a chronic disability of the wrist were analysed.

We calculated that we needed 50 patients with a chronic impairment of the wrist for the reliability analysis. For this analysis patients were recruited by going through lists of outpatient visits from 2005 to 2007 and identifying patients who had undergone treatment for wrist injuries during that particular period. The patients all suffered from any degree of chronic impairment of the wrist and had not received any active treatment during the past 12 months. In order to compensate for drop-outs and reluctance to participate, we aimed at including 20% patients more than needed. Patients were contacted by a research nurse by telephone and 62 patients who accepted participation in the study on the phone were mailed a letter including the DASH and the PRWE questionnaires, a consent letter, and a prepaid reply envelope. One week after the return of the first questionnaires, a second letter with the DASH and the PRWE questionnaires was mailed to the patient. Mean time between answering the two questionnaires was 17 days (range 5-174, median 14 days). The patients' medical charts were reviewed and information about the type of injury, date of injury, employment status and presence of other diseases was extracted from medical records. A majority of the respondents were women who had been treated for a distal radius fracture (Table 1). These patients are onward referred to as patients with a chronic impairment of the wrist.

**Table 1:** Demographic and injury data at baseline data on all patients included (n = 124)

	Patients with chronic wrist disability (n = 62)		Patients with an acute wrist injury (n = 62)	
Age at injury, yrs, mean; *SD *(range)	62; *13 *(26-85)		59; *16 *(15-88)	
Female, n (%)	52 (84)		50 (81)	
**Type of employment **				
Office work	21	32	15	23
Healthcare, physiotherapy or care of children	8	13	14	23
Retired	15	24	22	35
Other*	3	7	2	4
Missing	15	24	9	15
**Type of injury**				
Distal radius fracture	55	88	58	94
Distal radius and ulna fracture	1	2	2	3
Other injury hand/wrist**	6	10	2	3
**Type of treatment**				
Plaster cast	34	55	36	58
External fixation	13	21	17	27
Open reduction, internal fixation	5	8	6	10
Osteotomy	7	11	2	3
Other***	3	5	1	2

* air stewardess (n = 1), gym teacher (n = 1), bus driver (n = 1), manual labor (n = 2)

**scaphoid fracture (n = 1), fracture collum radii (n = 1), DRF + phalange fracture (n = 2), metacarpal fracture (n = 2), distal radius fracture + EPL-rupture (n = 2)

*** open reduction and pin fixation (n = 1,) EIP to EPL transfer (n = 2), external fixator and volar plate (n = 1)

### Validity

Validity is defined as the ability of an instrument to measure what it is intended to measure [17]. Four forms of validity were evaluated, viz. *face validity*, *content validity, discriminant validity*, and *criterion validity*.

#### Face validity

Face validity refers to how relevant the test appears to be from the point of view of the respondent. Face validity was tested by handing out the questionnaire to 18 healthy individuals working at our department. Thereafter, the questionnaire was handed out to 32 consecutive patients with a variety of orthopaedic injuries paying visit to our out-patient clinic.

#### Content validity

Content validity pertains to how well a test (e.g. items in an index) can be thought to measure the dimension it is intended to measure. Content validity was validated by an expert group consisting of three orthopeadic surgeons, one methodologist, and one research nurse.

#### Discriminant validity

Discriminant validity refers to the ability of a test to discriminate between different constructs or conditions. In this study discriminant validity was evaluated by comparing mean values from the PRWE total score for patients with chronic wrist disability compared to patients with an acute wrist injury.

#### Criterion validity

Criterion validity is a measurement to evaluate the correlation of an instrument to a pre-existing gold standard. DASH was in the present study considered to be the gold standard in evaluating wrist disease. Criterion validity was assessed by testing the predefined hypothesis concerning the expected relationship between the PRWE and DASH questionnaires. The result of the PRWE was expected to be approximately the same as the result of the DASH questionnaire. This analysis was performed on data from two groups of patients: patients with chronic wrist disability and patients with an acute injury. In total 115 patients who had filled in the DASH and the PRWE completely on the first occasion and 112 patients who had filled in the DASH and the PRWE completely on the second occasion were analyzed. Spearman's rho was used to calculate the correspondence between the PRWE and the DASH scores.

### Responsiveness

Responsiveness measures the ability of the questionnaire to detect clinical change. Responsiveness was assessed by comparing the PRWE values at baseline and at follow-up in a group of patients with an acute injury to the wrist. Responsiveness was assessed with the aid of standardized response mean (SRM) statistics, i.e. the observed mean change divided by the standard deviation of the observed change. The SRM is regarded as large (>0.8), moderate (0.5-0.8), or small (<0.5) [18].

We calculated that we needed 50 patients with an acute injury for the responsiveness analysis and expected 20% drop-outs or missing values. This second group of patients had recently been treated with a plaster cast or surgery due to an injury of the wrist or hand (Table 1). These patients were expected to have a clinically relevant improvement of their function, i.e. to have higher/worse PRWE scores at baseline compared to follow-up. A research nurse contacted the patients during their visit in the out-patient clinic during their clinical follow-up for their wrist injury. Sixty-two patients agreed to be included and gave their written consent. All patients were asked to complete the DASH and the PRWE questionnaires at 1 week and at 5 weeks after the removal of an external fixator or a plaster cast (mean time between measurements was 30 days, (range 13 - 64). The patients' medical charts were reviewed and information about the type of injury, date of injury, employment status, and presence of other disease was extracted. This latter group is onward referred to as the group with an acute injury to the wrist.

### Statistics

All statistics were extracted from SPSS 17.0. All tests were two-sided and 95% confidence intervals were calculated. The results were considered significant at p < 0.05. For the PRWE, only questionnaires with no more than 2 missing values in the pain subscore (of a total of 5 items) and no more than 4 missing values in the function subscore (of a total of 10 items) were accepted [12]. For the DASH questionnaire, only 3 missing items out of 30 were accepted [2]. Missing values for each scale within the limits above were replaced by the mean value of the responding items within each scale. Some patients were included in the analysis if only the PRWE score was necessary but excluded when a correlation between the DASH and the PRWE score was calculated.

## Results

The patients in our study were predominantly female and the mean age was 61 years of age and >90% had experienced a fracture in the wrist or distal fore-arm (Table 1). The results of the DASH and the PRWE at baseline and at the second measurement point did not significantly differ within the group with a chronic disease of the wrist. In the group with patients undergoing rehabilitation after a recent injury, the results of both the PRWE and the DASH improved markedly between the two measurements (Table 2). Floor and ceiling values are presented in Table 3.

**Table 2:** Total scores for the PRWE and DASH questionnaires at baseline (PRWE/DASH 1) and at follow-up (PRWE/DASH 2) for all patients (n = 124)

	Patients with chronic wrist disability (n = 62)	Patients with an acute wrist injury (n = 62)	Mean difference (Confidence interval)
	Mean; *SD*, (Range)	Mean; *SD, *(Range)	
**PRWE 1 total score**	19; *18 *(0 - 69)	52; *22 *(11 - 97)	-33 (-40; -26)***
**PRWE 2 total score**	22; *21 *(0 - 82)	30; *20 *(0 - 85)	-8 (-16; -1)*
**DASH 1 total score**	16; *14 *(0 - 52)	47; *21 *(3 - 93)	-31 (-37; -24)***
**DASH 2 total score**	17; *15 *(0 - 64)	27; *18 *(0 - 79)	-10 (-16; -3)**

* = p < 0.05; ** p < 0.01; *** p > 0.001

Mean differences and p-values calculated from t-test for independent samples

**Table 3:** Ceiling and floor effects of the PRWE and the DASH for all patients (n = 124)

	Pain subscore	Function subscore	PRWE total score	DASH
**Mean; *median *(range)**	17; *16 *(0-47)	18; *17 *(0-50)	35; *34 *(0-97)	31; *28 *(0-93)
**Floor effect % (n)**	15 (19)	12 (15)	12 (14)	7 (8)
**Ceiling effect % (n)**	0 (0)	2 (2)	0 (0)	0 (0)

### Reliability

#### Internal consistency

Cronbach's alpha coefficient calculated for the total score of the PRWE was 0.97, i.e. it showed an excellent internal consistency. For this analysis we used the total score of the PRWE for all patients at the first measurement point. We also analyzed the internal consistency for the pain and function PRWE subscales respectively. In this analysis both subscales had excellent internal consistency (Cronbach's alpha = 0.93 for the pain scale and 0.97 for the function scale).

#### Test-retest stability

The test-retest stability was assessed by using the intraclass correlation coefficient. The analysis was based on all complete questionnaires in the group with chronic impairment of the wrist (Table 4). The intraclass correlation coefficient was 0.93 on testing for the total score of the PRWE, thus showing excellent test-retest reliability. On analyzing all the complete DASH questionnaires in the chronic group, we found an intraclass coefficient of 0.93, which confirms that the PRWE is as reliable a tool for wrist evaluation as the DASH.

**Table 4:** Reliability (test-retest) expressed as intraclass correlation coefficient with 95% confidence intervals

	n	Baseline mean; *SD*	Follow-up mean; *SD*	Intraclass coefficient (95% confidence interval)
**Function subscore**	61	9; *10*	9; *10*	0.92 (0.87-0.95)
**Pain subscore**	61	11; *10*	12; *11*	0.89 (0.82-0.93)
**PRWE total score**	60	19; *18*	22; *21*	0.93 (0.88-0.96)
**DASH**	58	16; *14*	17; *15*	0.93 (0.89-0.96)

Results were calculated for patients with a chronic impairment of the wrist.

### Validity

#### Face validity

Face validity judged by the patients and healthy healthcare workers was good. None of the patients reported difficulties in understanding the content of the questionnaire. A few individuals made comments on the layout in the pain section of the questionnaire where they thought that the description of the scale could be left out. Some patients commented on the choice of words when describing "difficulty" in Swedish. We considered the Swedish words "svårighet" (difficulty), "problem" (problems) and "besvär" (trouble) and chose the latter. Some patients argued that the questions were not appropriate when the evaluation concerned the non-dominant hand.

#### Content validity

In the cultural adaptation process we noted that the question regarding "turning a door knob" evaluates the function in supination/pronation. In Sweden, door knobs are practically absent and doors are opened by pressing a door handle. We therefore changed the expression "turning a door knob" into "turning a tap or key", thereby describing the forced pronation or supination activity assessed with this item. In all other respect, the group found the content in the Swedish version of the PRWE no different than the original Canadian version of the PRWE. Thus, content validity was judged to be good by our expert group.

#### Discriminant validity

As visible from Table 2 the differences in mean values on the PRWE were large and statistically significant between patients with chronic wrist disability compared to patients with an acute wrist injury. Accordingly, the ability of the PRWE to discriminate between these patient groups was considered to be good.

#### Criterion validity

Criterion validity was assessed by calculating the correlation between the PRWE and the DASH scores. Spearman's rho was 0.92 (p < 0.001) at the first measurement point and 0.88 (p < 0.001) at the second measurement point, which confirms a good correlation between the two questionnaires.

### Responsiveness

Responsiveness was assessed with the aid of the standardized response mean (SRM). Responsiveness was calculated for the total score of the PRWE as well as for the separate subscales (Table 5). It was also calculated for the DASH score. In our series we had a good responsiveness with an SRM_PRWE _of 1.29 and an SRM_DASH _of 1.33. The observed clinical change between the first and the second measurement was statistically significant.

**Table 5:** Internal responsiveness statistics for the PRWE

	Baseline Mean; *SD*	Follow-up Mean; *SD*	Observed change^1 ^ Mean; *SD * (95% confidence interval)	*p*-value^2^	SRM
**Function (n = 58)**	28; *13*	14; *11*	14; *10 *(11-16)	<0.001	1.34
**Pain (n = 62)**	23; *11*	15; *11*	8; *9 *(6-10)	< 0.001	0.92
**PRWE total (n = 58)**	52; *22*	30; *21*	22; *17 *(18-27)	< 0.001	1.29
**DASH total (n = 53)**	47; *21*	27; *19*	21; *16 *(16-25)	< 0.001	1.33

The analysis was performed on patients with an acute wrist injury in whom a change in clinical status between the two measurements was expected.

**^1 ^**Observed change in results of questionnaire 1 week after removal of plaster cast or external fixator and 4-5 weeks later.

**^2 ^**p values given for differences between the two measurements described above. Paired samples t-test.

SRM = standardised response mean

### Analysis of missing values

A total of 248 PRWE and 248 DASH questionnaires from 124 patients were returned; 121 patients filled out the PRWE completely at the first occasion and 120 at the second occasion. The corresponding figures for the DASH questionnaire were 116 and 115, respectively. In summary, a total of 24 PRWE or DASH questionnaires were excluded due to missing values.

A detailed analysis of the 124 patients that filled out the PRWE questionnaire at the first occasion showed that 6 out of 124 patients had left out the question "cut meat using a knife in my affected hand", 8 out of 124 "carry a 10 lb object in my affected hand" and 7 out of 124 patients had left out the question "Work (your job or usual everyday work)". The patients' written comments why they had not answered these questions were for example "I am a vegetarian", "I only use my right wrist for such an activity", "my husband helps me with carrying heavy objects", "I have never had a job. All I did was to take care of the house and the children. Nowadays I only take care of the laundry, cooking and cleaning". Other missing values were evenly distributed with 0 to 3 missing values per item.

## Discussion

Our results showed that the PRWE is a self-administered questionnaire with good validity, reliability, as well as responsiveness and is therefore helpful in evaluating the results after treatment of a wrist injury or disease of the wrist.

When there is an accepted and validated upper extremity score such as the DASH, why bother to introduce a new instrument for evaluating wrist fractures? An advantage with the PRWE compared to the DASH is that it is shorter and easier to fill out. The DASH consists of as many as 30 items and has high demands for being considered completely filled out. To provide a shorter alternative, two versions of a Quick-DASH have been developed with 9 and 11 items respectively [19,20]. Their validity has been questioned [20] and further investigations of validity and precision of the Quick-DASH are needed before it can be widely recommended. As an alternative to the Quick-DASH, we believe the PRWE to be a very comprehensive and user-friendly wrist-specific evaluation score.

Another validation and translation of the PRWE to the Swedish language has recently been published by Wilcke et al [21]. Wilcke concluded that the PRWE is valid and responsive. The translation by Wilcke et al and our version differ slightly but are strikingly alike, considering that they have been constructed at two separate centers not aware of each other's works or intentions.

As described in the result section we changed the expression "turning a door knob" into "turning a tap or key". It was interesting to note that the same question was also modified by Wilcke et al [21]. Their choice of Swedish translation was "open a tight or new jar", which is also an item in the DASH-questionnaire [2] (both in the original and the validated Swedish version [5]). However, opening of a jar represents an adduction/abduction of the hand more than the forced pronation/supination described in the original version's door knob. We therefore consider our PRWE-Swe to be closer to the original version than the Wilcke version. Besides this minor difference between the two Swedish versions of the PRWE, no other differences were noted. Therefore, both of them should be considered as valid and reliable tools for assessing pain and disability of the wrist.

Changulani et al have questioned the validity of the PRWE [7]. They claimed that during the validation process of the PRWE, there was only a weak to moderate correlation between the PRWE and the impairment scores that were used as a reference tool. In the present study, however, we found an excellent correlation between the DASH and the PRWE, thereby confirming the criterion validity of the PRWE.

The Cronbach alpha calculated in the questionnaire resulted in a high value (0.93 on the pain subscale, 0.97 on the function subscale, and 0.97 for the total score) thus representing a high internal consistency i.e. that all questions of the questionnaire are investigating the same quality. One might argue that some of the questions could be left out since the total score would not change if one or more questions would be left out. However, due to the intention to keep this Swedish version of the PRWE as similar to the original PRWE as possible we kept the items unchanged.

The test-retest reliability of PRWE-Swe was found to be good with an intraclass coefficient corresponding to the findings of the original validation of the PRWE [13]. A difference with our test-retest reliability compared to the original validation is that we tested patients at two occasions rather close in time, whereas the original validation analyzed stability over a period of time of a year. These findings thus suggest that the test-retest stability of the PRWE is good both in a short and long perspective.

The difference in the total PRWE score for patients with a chronic wrist disability and with acute injury, respectively, confirms the discriminative ability of the PRWE. A large difference in absolute figures was noted between these two groups at the first measurement point. The PRWE questionnaire thus clearly reveals a difference in the degree of disability when comparing patients with a severe acute condition and a chronic low-grade impairment of the wrist. The relative difference between the groups was similar when comparing the PRWE and the DASH-figures.

While analysing the internal responsiveness in the group with a recent injury to the wrist, we found that the observed change in both the total PRWE score and the two subscores were significant, with large SRMs and large observed changes between the time for removal of plaster cast or external fixator and at 4-5 weeks later. The SRM for the PRWE total score and the DASH were of similar size indicating a comparable responsiveness for these two instruments. However, a limitation of this methodology is that the expected improvement over this period is rather large and it says nothing about responsiveness for more subtle clinical changes.

Our study provides analysis of the reliability and responsiveness of the two subscores of the PRWE and confirms MacDermid's opinion that these subscales are in themselves reliable and with good responsiveness [13].

When making the analysis of the responsiveness of the pain and function subscales of the PRWE-Swe we found that the index for function changed more than the index for pain. This suggests that the rehabilitation process after a wrist injury is painful although the functional loss soon improves. It would be interesting to follow a group of patients with various diseases and degrees of disability of the wrist. If performed with repeated measurements in the acute and the long term perspective the discriminative ability and the responsiveness of the PRWE could be studied in more detail.

We assume that mailing questionnaires may have resulted more often in missing values than would have been the case if we had asked the patients to fill in the questionnaire in our presence. However, there were only a few missing values, which confirms that the PRWE is user-friendly. The missing values in the PRWE were mainly concerning the items of cutting meat, carrying a 10 lb object and work activities. Most of the respondents were women and many were retired. This may explain why the item for carrying 10 lb object resulted in several missing values. Furthermore, it enlightens the lack of consideration taken for handedness in the PRWE. Many of the questions in the function subscale of the PRWE are only relevant if the patient has injured the dominant hand. Some patients left out questions concerning such items as "cut meat using my affected hand" with explanations such as "I only use my right wrist for such an activity". The DASH asks the patient to rate their ability irrespective of which hand that is injured whereas the PRWE asks how well the patient performs with the injured hand. One could expect the DASH to be less sensitive to handedness. As we have shown, however, the results of the PRWE and the DASH are very similar and the instructions to the patients do not seem to affect the result of the questionnaire. This finding is supported by another study [22] that shows that injury to a dominant shoulder affects the DASH-score in a proportionate way whereas injury to the non-dominant shoulder does not affect the DASH-score in an expected manner. An optimal upper extremity score should have a way of handling the fact that handedness strongly affects the outcome of the questionnaire. This is a weakness of the PRWE and the DASH in their role as instruments for evaluation of wrist function.

A limitation of our study is that the vast majority of respondents were women and the mean age was rather high. It would have been desirable to include more men and more young people to ensure the general applicability of our results. Furthermore, the respondents only represent the patients willing to participate in our investigation, thus introducing selection bias in our study.

## Conclusions

We conclude that the PRWE-Swe is a reliable and valid instrument for evaluating the function of the wrist. The PRWE-Swe is offered as a useful tool for measuring outcome in future clinical studies. It is suitable as a follow-up instrument for professionals in clinical practice. The use of the PRWE-Swe could also be broadened to serve as a framework for insurance companies to assess any remaining disability after wrist injury.

## Competing interests

The authors declare that they have no competing interests.

## Authors' contributions

CM designed the study, translated the PRWE questionnaire, constructed the SPSS file, performed the statistical analyses, and wrote the manuscript. GB counselled during the translation process, performed the statistical analyses, and reviewed the manuscript. SP took the initiative to do the study and reviewed the manuscript. LA supervised the study design and the adaptation process and reviewed the manuscript. HT contributed clinical expertise during the translation process and reviewed the manuscript. All authors read and approved the final manuscript.

## Pre-publication history

The pre-publication history for this paper can be accessed here:

http://www.biomedcentral.com/1471-2474/12/171/prepub

## Supplementary Material

Additional file 1 **The PRWE-Swe questionnaire**. The Additional File 1 (Adobe Acrobat Document, 49 kB) contains the questionnaire that has been evaluated in this study. Click here for file
