# Supplementary material for: Measuring Outcome after Wrist Injury: Translation and Validation of the Swedish Version of the Patient-Rated Wrist Evaluation (PRWE-Swe)
Source: BMC Musculoskelet Disord. 2011 Jul 22;12:171. doi: 10.1186/1471-2474-12-171 (PMC3152541; doi:10.1186/1471-2474-12-171)
Supplement: Additional file 1 — The PRWE-Swe questionnaire. The Additional File 1 (Adobe Acrobat Document, 49 kB) contains the questionnaire that has been evaluated in this study. [file 1471-2474-12-171-S1.PDF]

HÄLSOENKÄT - HANDLED

Namn: ______________________ Datum: __________

Personnummer: ______________________

*Nedanstående frågor hjälper oss att förstå hur mycket **besvär** du har haft av din handled den **senaste veckan**. Du ska beskriva ett **genomsnitt** av dina **symptom** från handleden under den **senaste veckan** på en skala från 0 till 10. Var snäll och besvara **alla** frågor. Om du inte utförde någon av aktiviteterna så försök att **uppskatta** den **smärta** eller **svårighet** du kunde förväntat dig. Om du aldrig utfört någon av aktiviteterna kan du låta bli att svara.*

SMÄRTA

*Uppskatta den **genomsnittliga graden av smärta** i din handled den **senaste veckan** genom att ringa in den siffra som bäst beskriver din smärta på en skala från 0 till 10.*

*Noll (**0**) betyder att du inte hade någon smärta och tio (**10**) betyder att du hade den värsta smärta du någonsin känt eller att du inte kunde utföra aktiviteten pga smärta.*

	**Ingen smärta**										**Värsta tänkbara smärta**
I vila	0	1	2	3	4	5	6	7	8	9	10
När du utför en uppgift med upprepad handledsrörelse	0	1	2	3	4	5	6	7	8	9	10
När du lyfter ett tungt föremål	0	1	2	3	4	5	6	7	8	9	10
När det är som värst	0	1	2	3	4	5	6	7	8	9	10
	**Aldrig**										**Alltid**
Hur ofta har du ont?	0	1	2	3	4	5	6	7	8	9	10

FUNKTION

*Uppskatta **graden av besvär** du upplevde för var och en av nedanstående aktiviteter under den **senaste veckan** genom att ringa in den siffra som motsvarar dina besvär på en skala från 0 till 10. Noll (**0**) betyder att du inte hade några svårigheter och tio (**10**) att det var så svårt att du inte alls kunde utföra aktiviteten.*

**Specifika aktiviteter**	**Inga besvär**										**Omöjligt att utföra**
Vrida om en kran eller nyckel med din drabbade hand	0	1	2	3	4	5	6	7	8	9	10
Skära kött med kniv med din drabbade hand	0	1	2	3	4	5	6	7	8	9	10
Knäppa knappar i din skjorta/blus	0	1	2	3	4	5	6	7	8	9	10
Använda din drabbade hand för att resa dig ur en stol	0	1	2	3	4	5	6	7	8	9	10
Bära ett 5 kg tungt föremål med din drabbade hand	0	1	2	3	4	5	6	7	8	9	10
Använda toalettpapper med din drabbade hand	0	1	2	3	4	5	6	7	8	9	10

**Allmänna aktiviteter**	**Inga besvär**										**Omöjligt att utföra**
Personlig vård (klä på dig, tvätta dig)	0	1	2	3	4	5	6	7	8	9	10
Hushållsarbete (städning, underhåll)	0	1	2	3	4	5	6	7	8	9	10
Arbete (ditt yrkesarbete eller dina vardagliga uppgifter)	0	1	2	3	4	5	6	7	8	9	10
Fritidsaktiviteter	0	1	2	3	4	5	6	7	8	9	10
